# Dr. Wolff’s Criticism

**Published:** 1888-02

**Authors:** 


DR. WOLFF'S CRITICISM.—The Doctor has erected a windmill of his own construction, and charges it most valliantly. He misquotes our quotation of Dr. Thorne's excellent article; while the assumption that we are opposed to quarantine, in general, and that we make war on the Texas quarantine in particular, is entirely gratutious; there is nothing in the offending "leader," to warrant it. In all essentials Dr. Wolff's views, and our own, agree; and especially when he says that "what Texas needs and must have"—and upon which "the safety of the people depends"—is "a rigid quarantine, with an inspection, isolation, disinfection, etc.," for that is our idea, exactly, and he has expressed it in our very words, (omitting only, "non-detention of the *well*). We do not propose isolation and inspection, (with "*non-detention of the well*") in lieu of quarantine, but in addition thereto--and it was this plan—"the only plan, which experience had demonstrated to be efficient" that we urged should be put into operation in New York, releasing, at the same time, the well persons who had not been exposed, and those who had been exposed, after a period of observation. Our understanding is, that Dr. Thorne's idea and the "English plan" are *not* isolation and inspection with non-detention of the well *in lieu of* quarantine, but that it is modified quarantine—quarantine of sick and infected—or quarantine *plus* these things.

With regard to Dr. Smith, Dr. Wolff seems to forgets that we said "we have no knowledge of what Dr. Smith is doing, but *assume* from his well-known reputation, etc.," that he is putting into execution all the modern ideas, etc. (words to that effect), and that "*if it should transpire* that he is willfully or ignorantly shutting his eyes, etc., he deserved the condemnation,"—which, Dr. Wolff says he has received at the hands of ever-so-many corporations.—

But if our offensive "leader" should "lead" to no other results, it is paid for by the interesting paper of our distinguished friend, the able and zealous Cerberus, who guards the gate at Brazos, Dr. A. S. Wolff.

